
JOURNAL INFORMATION
==============================
NLM Title Abbreviation: Tex Med J (Austin)
NLM Journal ID: 21130590R

Texas Medical Journal

ISSN: 0892-8495
Publisher: Biomedical Journal Digitization Project

ARTICLE INFORMATION
==============================
PMCID: PMC9590985
Article version: 1
Subjects: Books and Magazines

A Treatise on the Science and Practice of Midwifery

Print publication date: 1899 Apr
Volume: 14
Issue: 10
First page: 597
Last page: 597
License: This work is free of known copyright restrictions. For more information, please see PMC Back Issue Digitization.
License URL: https://creativecommons.org/publicdomain/mark/1.0/

==============================

---

A Treatise on the Science and Practice of Midwifery.—By W. S. Playfair, M. D., LL. D., F. R. C. P., Emeritus Professor of Obstetric Medicine in King's College, London; Examiner in Midwifery to the Universities of Cambridge and London; Seventh American from the Ninth English Edition. In one very handsome octavo volume of 700 pages, with 207 engravings and seven full-page plates. Cloth, $3.75, net. Leather, $4.75, net. Lea Brothers & Co., Publishers, Philadelphia and New York.

Playfair is as familiar to the profession as household words, and nothing need be said as to its general merits. It has now attained its majority, having been written in 1876. During this time nine English and seven American editions have been called for. No work on any medical subject is so widely known, with the exception of Gray's Anatomy and the United States Dispensatory. Its gifted author still lives, and retains his position at the head of his speciality, and has taken unusual pains to write into the present edition all the recent progress in obstetrical art. He who purchases this last edition of Playfair can have the assurance that he has the best which this or any other language affords. I. J. J.

---

### Publisher's Notes.

In view of the fact that cod-liver oil in its crude, refined or emulsified form indicates atony of the digestive organs and hypertrophy of the gastric and intestinal mucous membranes, it is evident that the easily digestible and assimilable Hagee's Cordial, containing as it does the true active principles of the oil, is the most eligible succedaneum for this oil.

---

Removal Notice.—On April 1, 1899, we will remove our business from our present location to our commodious new factory building, Nos. 42, 44, and 46 Germania Avenue, Jersey City, N. J.
